# Long noncoding RNA ZFPM2-AS1 acts as a miRNA sponge and promotes cell invasion through regulation of miR-139/GDF10 in hepatocellular carcinoma

**DOI:** 10.1186/s13046-020-01664-1

**Published:** 2020-08-14

**Authors:** Hui He, Yawei Wang, Peng Ye, Dehui Yi, Ying Cheng, Haibo Tang, Zhi Zhu, Xun Wang, Shi Jin

**Affiliations:** 1 Department of Laparoscopic Surgery, the First Affiliated Hospital of Dalian Medical University, Dalian, 116000 Liaoning Province China; 2 Department of thoracic surgery, Cancer Hospital of China Medical University, Liaoning Cancer Hospital& Institute, Shenyang, 110042 Liaoning Province China; 3 Department of Urological Surgery, Cancer Hospital of China Medical University, Liaoning Cancer Hospital & Institute, Shenyang, 110042 Liaoning Province China; 4 Department of organ transplantation& hepatobiliary surgery, the First Affiliated Hospital of China Medical University, Shenyang, 110042 Liaoning Province China; 5 Department of Gastrointestinal & Hernia & Bariatric Surgery, the Third Xiangya Hospital of Central South University, Changsha, 410000 Hunan Province China

**Keywords:** LncRNA ZFPM2-AS1, miR-139, Liver hepatocellular carcinoma, TCGA, Bioinformatics analysis

## Abstract

**Background:**

Emerging evidence has shown that dysregulated expression of long noncoding RNAs (lncRNAs) is implicated in liver hepatocellular carcinoma (HCC). However, the role and molecular mechanism of differentially expressed lncRNAs in HCC has not been fully explained.

**Methods:**

The expression profiles of lncRNAs in HCC samples were derived from microarrays analysis or downloaded from The Cancer Genome Atlas (TCGA), and their correlation with prognosis and clinical characteristics were further analyzed. Silencing of lncRNA ZFPM2-AS1 was conducted to assess the effect of ZFPM2-AS1 in vitro. The miRcode and Target Scan databases were used to determine the lncRNA-miRNA-mRNA interactions. The biological functions were demonstrated by luciferase reporter assay, western blotting, PCR and rescue experiments.

**Results:**

The expression level of lncRNA ZFPM2-AS1 was significantly higher in HCC tissues than in adjacent normal tissues, and higher ZFPM2-AS1 was remarkably related to poor survival. Functionally, silencing of lncRNA ZFPM2-AS1 inhibited cell proliferation, migration, invasion and promoted cell apoptosis in vitro. Bioinformatics analysis based on the miRcode and TargetScan databases showed that lncRNA ZFPM2-AS1 regulated GDF10 expression by competitively binding to miR-139. miR-139 and downregulated GDF10 reversed cell phenotypes caused by lncRNA ZFPM2-AS1 by rescue analysis.

**Conclusions:**

ZFPM2-AS1, an upregulated lncRNA in HCC, was associated with malignant tumor phenotypes and worse patient survival. ZFPM2-AS1 regulated the progression of HCC by acting as a competing endogenous RNA (ceRNA) to competitively bind to miR-139 and regulate GDF10 expression. Our study provides new insight into the posttranscriptional regulation mechanism of lncRNA ZFPM2-AS1 and suggests that ZFPM2-AS1/miR-139/GDF10 may act as a potential therapeutic target and prognostic biomarker for HCC.

## Background

Liver hepatocellular carcinoma (HCC) is the fifth most common malignancy and the third leading cause of cancer-related death worldwide [1]. The occurrence of HCC shows an increasing trend around the world, especially in China [2]. Surgical resection, liver transplantation and tumor ablation are potentially curative therapies [3–5]; however, these treatment options are only applicable to patients with early stage disease [6, 7]. In recent years, more research has focused on targeted therapy for HCC. Therefore, there is an urgent need to study in depth the main targets of hepatocarcinogenesis development and tumor metastasis.

Recently, high-throughput genome and transcriptome sequencing and microarrays have indicated that apart from protein-coding genes, 75% of the human genomeis transcribed into noncoding RNAs [8]. LncRNAs are functionally catalogued as noncoding transcripts, are more than 200 nucleotides in length, and have no protein-coding potential. Aberrant expression and deficiency or mutations of lncRNAs have been reported to be involved in numerous complex diseases, including cancers [9, 10]. Mounting evidence has indicated that lncRNAs are implicated in a variety of biological processes, including chromatin interaction, transcriptional regulation, mRNA posttranscriptional regulation and epigenetic regulation [11–13].

Additionally, increasing experimental evidence supports the notion that lncRNAs function as competitive endogenous RNAs (ceRNAs), which compete for microRNA (miRNA) binding to upregulate the expression of target genes [14]. The ceRNA hypothesis has provided new insights into the function of a large amount of novel lncRNAs. For example, some studies have shown that the expression of pseudogene RP11-424C20.2 and its parental gene UHRF1 in HCC and thymoma is frequently up-regulated, and there is a significant positive correlation. It was further found that RP11-424C20.2, as a competitive endogenous RNA (ceRNA), may increase the expression of UHRF1 in response to mir-378a-3p, and that there is a strong correlation between UHRF1 and immune related biological processes through functional enrichment analysis [15]. Moreover, it has been reported that lncRNA HOTAIR regulates notch3 expression by sponging miR-613 in pancreatic cancer [16].

Thus, we hope that bioinformatics analysis can identify a lncRNA-microRNA-mRNA axis that can influence the progression of hepatocellular carcinoma, providing a new direction for targeted therapy of HCC. In this study, we used the TCGA database to identify a significant lncRNA strongly associated with clinical prognosis, and then the downstream miRNA and mRNA were predicted based on the miRcode [17] and Target Scan databases. Finally, to determine biological functions of the identified lncRNA-microRNA-mRNA in HCC we conducted a series of experiments both in vivo and in vitro.

## Materials and methods

### Microarrays and computational analysis

Sample preparation and microarray hybridization were performed by Kangchen Bio-tech, (Shanghai P.R. China). Four paired fresh poorly differentiated HCC tissues and corresponding noncancerous tissues were used for microarray analysis (Arraystar Human LncRNA Microarray V4.0 is designed for the global profiling of human LncRNAs and protein-coding transcripts, Table S1). Briefly, total RNA was extracted with TRIzol® Reagent (Invitrogen, Carlsbad, CA, USA) and purified using the RNeasy Mini Kit (Qiagen, Valencia, CA, USA). cDNA was synthesized and labeled before it was purified and hybridized to the microarray (Arraystar, Rockville, MD, USA).

### TCGA data downloading and preprocessing and screening of differentially expressed lncRNAs

RNAseq expression profile data for HCC were downloaded from the TCGA database. The RNAseq data were combined, and the gene symbols were converted using a Perl script to isolate lncRNAs. There were 425 samples in the data set, among which 50 matched samples were selected according to the paired sample number for screening differentially expressed lncRNAs (Table S2). The data were normalized, and differentially expressed lncRNAs were screened using the EdgeR [18] package in R software. The screening criteria were *P* value< 0.05 and |logFC| > 2. The upregulated lncRNAs and downregulated lncRNAs obtained by the differential analysis from the TCGA database were combined with the significantly different ones obtained from our own experimental chip analysis (Table S3), and a Venn diagram was generated using R software. Survival data from the TCGA database was downloaded, extracted and cross-referenced with the expression values in all samples of the obtained lncRNAs with common co-upregulation or co-downregulation using Perl language, and each sample was assigned the expression value of lncRNAs. The survival data were combined, and then the R survival package was used for OS batch analysis to screen for differentially expressed lncRNAs. *P* < 0.05 was considered to be significant.

### Cell culture and transfection

Huh7 and HCCLM3 liver cancer cells were obtained from the Shanghai Cell Bank (Shanghai, China). Huh7 cells were cultured in RPMI-1640 (Invitrogen, Carlsbad, CA). HCCLM3 cells were grown in DMEM (Invitrogen). All media were supplemented with 10% fetal calf serum (Invitrogen) and 100 IU/ml penicillin (Sigma, St. Louis, MO). shRNAs specifically targeting ZFPM2-AS1 were constructed and synthesized by RiboBio (Guangzhou, China). miR-139 mimic and miR negative control were provided by Gene Pharma (Shanghai, China).

For GDF10 overexpression, full-length human GDF10 cDNA was amplified by PCR and subcloned into the mammalian expression vector pcDNA3.1(+) (Invitrogen). The empty vector was used as a control. For cell transfection, cells were dissociated into a single cell suspension and plated in 6-well plates at a density of 5 × 10^5^ cells/well. Lipofectamine 2000 Reagent (Invitrogen) was used for transfections. At 48 h posttransfection, cells were harvested for further analysis.

### RNA extraction and RT-qPCR analysis

Total RNA was isolated from the prepared tissues and cells using TRIzol Reagent (Invitrogen). Complementary DNA was synthesized using the PrimeScript RT reagent Kit (TaKaRa, Dalian, China). QPCR was then performed using the fluorescent dye SYBR Green Master Mix (Applied Biosystems, Foster City, CA, USA) on the ABI PRISM 7900 Sequence Detection System (Applied Biosystems). The data were calculated using the 2^−ΔΔCt^ method, and GAPDH was used as an endogenous control. The primers were designed as follows:

ZFPM2-AS1: forward 5′- CAATGGGACTAAGCCAGGCA-3′,

ZFPM2-AS1: reverse 5′- GGGCTCCACCAACAACCATA-3′;

si-ZFPM2-AS1: ATGAATTTAACTCACTAATTTCA.

GAPDH: forward, 5′-ATAGCACAGCCTGGATAGCAACGTAC-3′,

GAPDH, reverse, 5′-CACCTTCTACAATGAGCTGCGTGTG-3′.

### Cell proliferation, migration, invasion, apoptosis and cell cycle analysis

The Cell Counting Kit 8 was used to measure cell viability. The spectrophotometric absorbance at 450 nm for each sample was detected using an Infinite M200 spectrophotometer (Tecan). All experiments were repeated three times with six replicates. The transwell assay was used to evaluate cell migration. Cell invasion was assessed using the BioCoat Matrigel Invasion Chamber (BD Biosciences). Cell numbers for cell migration and invasion were counted in three random fields. Cells were stained with Annexin V and propidium iodide using the Annexin V–FITC Apoptosis Detection kit (Invitrogen), and the percentage of apoptotic cells was examined with flow cytometry (Beckman, USA). For detection of the cell cycle, cells were stained with PI after 48 h of transfection and were examined by FACS.

### Colony formation assay

HCC cells were seeded in 6-well plates, cultured in complete medium for 24 h, and cultured in a medium containing a final concentration of 1 mg/ml G418 (formulated as a 400 mg/ml stock solution) to observe any obvious cell clonal masses. After 14 days, the culture solution in the 6-well plate was discarded and washed twice with precooled PB. Then, 95 μl of 95% ethanol was added to each well, and the cells were fixed at room temperature for 10 min. When white spots on the bottom of the plate appeared, the fixing solution was discarded, and the cells were washed twice with PBS. Then, 0.1 mM crystal violet (500 μl) was added to each well, stained for 10 min, discarded, washed twice with PBS, and the results were analyzed under a microscope.

### Luciferase reporter assay

The wild-type miR-139-wt and GDF10 3′-UTR-wt (… ACUGUAG …) and the corresponding mutant miR-139-mut and GDF10 3’UTR-mut (… CACTCAT …) were cloned into the pMIR-REPORT Luciferase plasmid vector into the MluIand HindIII restriction endonuclease sites upstream and downstream, respectively.

Huh7 and HCCLM3 cells were seeded in 96-well plates, and the pcDNA/pre-miR-139 vector was separately ligated with pMIR-ZFPM2-AS1 and pMIR- miR-139-mut /wt using Lipofectamine 2000. The luciferase activity was detected by co transfection of pMIR-ZFPM2-AS1, pMIR- miR-139-mut/wt vector for 48 h. The luciferase activity was assessed by the Dual-Luciferase Reporter Assay System (Promega, Madison, WI, USA), and firefly luciferase activity was normalized to Renilla luciferase activity.

### RNA immunoprecipitation (RIP) assay and RNA pull-down

RIP was performed using a Magna RNA-binding protein immunoprecipitation kit (Millipore, Bedford, MA) according to the manufacturer’s instructions. Briefly, cell lysates were incubated with RIP buffer containing magnetic beads conjugated with negative control normal IgG or Anti-Ago2-ChIP Grade (Abcam, No. ab32381). The samples were then incubated with Proteinase K to isolate the immunoprecipitated RNA. Finally, purified RNAs were extracted and subjected to PCR and real-time PCR to confirm the presence of the binding targets (lncRNA ZFPM2-AS1 & miR-139).

The non-specific and specific biotin labeling hybridization probe of miR-139 and ZFPM2-AS1 were designed and synthesized by Sangon company (Shanghai, China). On the first day, cells were cross-linked with paraformaldehyde, lysed, and sonicated before the hybridization step that was performed by adding biotinylated specific probes. Magnetic streptavidin beads (Solarbio, Beijing, China) were then added to separate specific material from the cell lysate. On the second day, beads were isolated by a magnet and washed five times. A de-crosslinking step allowed recovery of RNAs that were purified and used for qRT-PCR analysis.

### Western blot analysis

Cell samples were lysed in radio immunoprecipitation assay lysis buffer (Beyotime, Shanghai, China). The protein concentration was determined by the Enhanced BCA Protein Assay Kit (Beyotime), and equal amounts of protein were separated by SDS-polyacrylamide gel electrophoresis and transferred to polyvinylidene difluoride (PVDF) membranes (Millipore, Bedford, MA, USA). After blocking with 5% nonfat milk for 1 h, the membranes were incubated with the specific primary antibodies overnight at 4 °C. Then, the membranes were washed three times with TBST and incubated with horseradish peroxidase (HRP)-conjugated secondary antibodies for 2 h at room temperature. Protein bands were visualized using an enhanced chemiluminescence (ECL) system (Pierce Biotechnology Inc., Rockford, IL, USA) and analyzed with AlphaImager2200 image software (UVP, Upland, CA, USA). GAPDH (1:1000, Sigma, St. Louis, MO, USA) was used as a loading control.

### Nude mouse xenograft model

The experimental animals were 6-week-old BALB/c nude mice. The HCC cells of each experimental treatment group were washed, suspended, and 1 × 10^7^ cells/0.1 ml single cell suspension were inoculated into the armpits of nude mice. The long diameter (a) and short diameter (b) of the subcutaneous xenograft tumors were measured weekly. The tumor volume was calculated according to the formula V = ab^2^/2, and growth curves of the tumors were generated. After 6 weeks, the nude mice were sacrificed and the tumors were extracted. The tumors were weighed, and pictures were taken.

### Statistical analysis

All statistical analyses were conducted using GraphPad Prism 6.0 software (GraphPad Software, San Diego, CA, USA) and SPSS version 18.0 software (SPSS Inc., Chicago, IL, USA). Each experiment is representative of at least three independent experiments. The data are presented as the mean ± standard deviation (SD). Differences between experimental groups were assessed by Student’s t-test or one-way ANOVA. The association between ZFPM2-AS1 expression and the clinicopathological features of HCC patients was evaluated using the chi-square test. Survival curves were plotted using the Kaplan–Meier method and were compared with the log-rank test. Two-sided *P*-values were calculated, and a probability level of 0.05 was considered statistically significant.

## Results

### Data download and differential expressed lncRNA screening

**Fig. 1:**
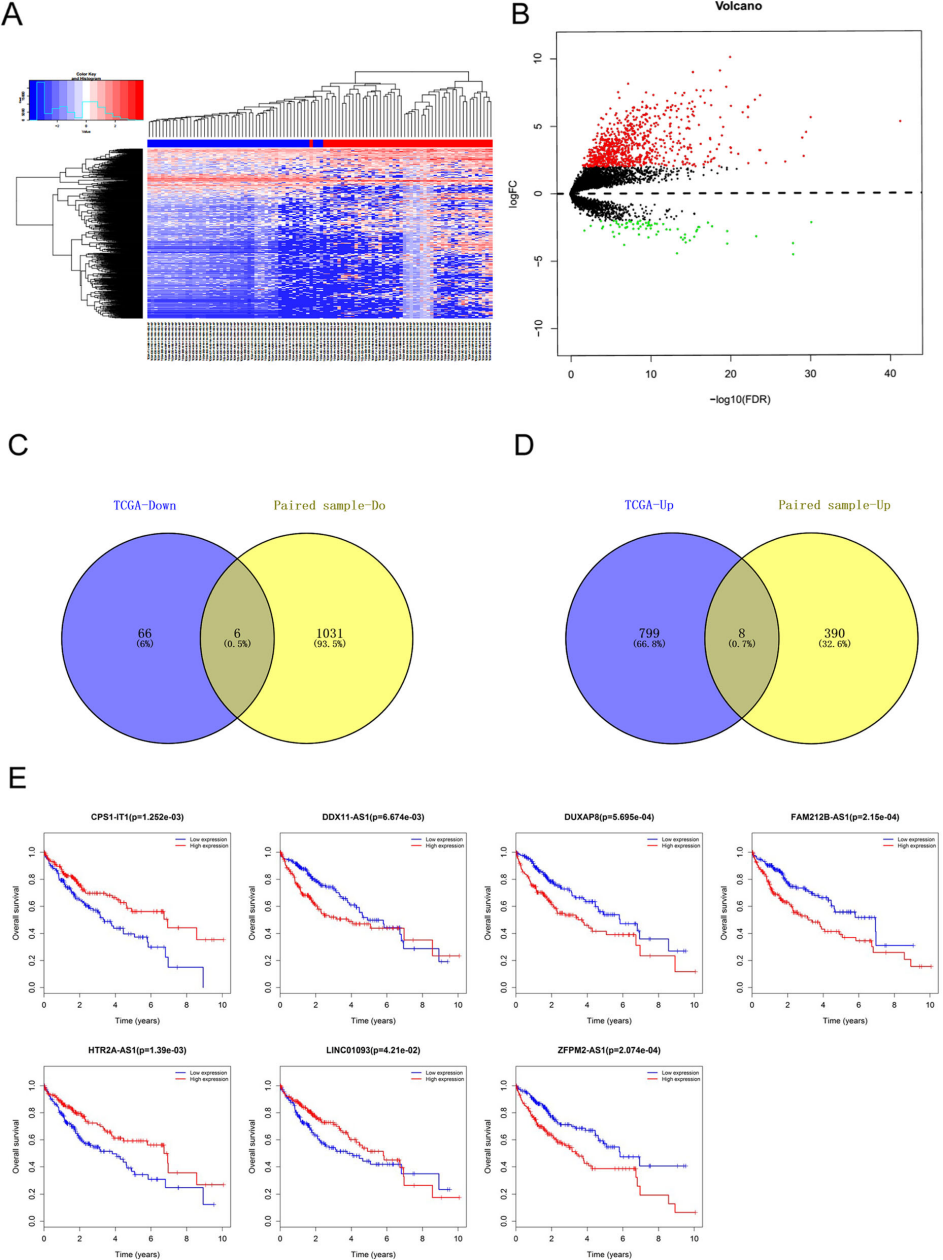
Data download and differential expressed lncRNA screening for HCC. **a** The heat map of differentially expressed lncRNAs for TCGA samples. **b** The volcano map of differentially expressed lncRNAs for TCGA samples. **c** Up regulated overlapping differentially expressed lncRNAs for HCC samples. **d** Downregulated overlapping differentially expressed lncRNAs for HCC samples. **e** The correlation between differentially expressed lncRNA and OS of HCC patients

A total of 425 liver cancer samples were downloaded from TCGA, of which 50 were normal paracancerous paired samples. After differential analysis, a total of 879 differentially expressed lncRNAs were obtained, of which 807 were upregulated and 72 were downregulated. The differential analysis heat map and volcano plot are shown in Fig. 1a and b.

When the 807 differentially upregulated TCGA lncRNAs were cross-referenced with 398 lncRNAs upregulated in the experimental sample, a total of 8 overlapping lncRNAs were identified, as shown in Fig. 1c Between the 72 differentially downregulated TCGA lncRNAs and the 1037 lncRNAs that were downregulated in the experimental samples there were 6 overlapping lncRNAs, as shown in Fig. 1d, which totaled 14 overlapping genes.

The results of the OS batch survival analysis of the overlapping lncRNAs showed that 7 lncRNAs were significantly correlated with OS, and the main lncRNAs are shown in Fig. 1e.

### Characterization of lncRNA ZFPM2-AS1 in HCC

**Fig. 2:**
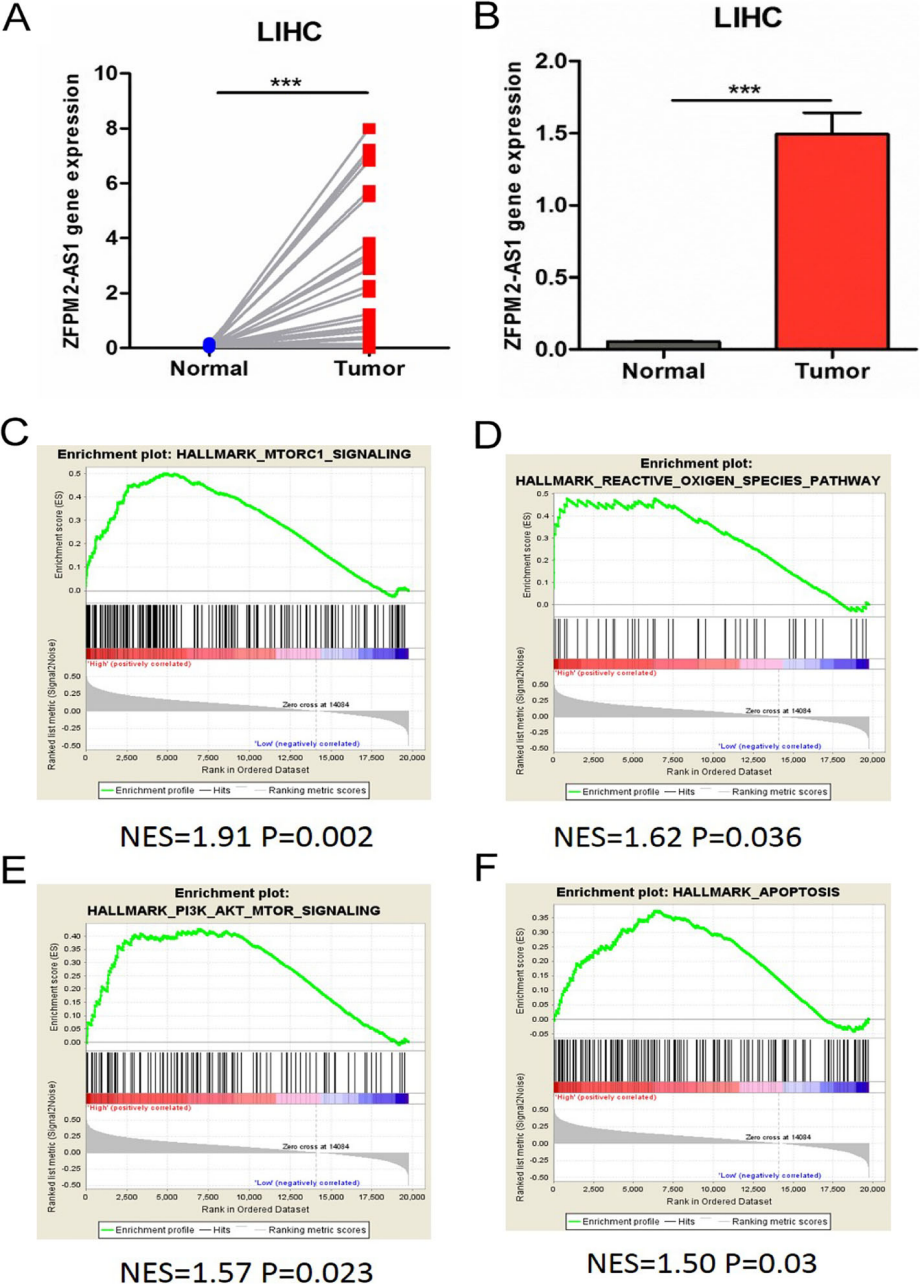
ZFPM2-AS1 is upregulated in HCC tissues. **a** The expression of ZFPM2-AS1 in paired HCC specimens and corresponding adjacent non tumor tissues. **b** The mean expression level of ZFPM2-AS1 in tumor and para-carcinoma tissue for HCC patients. **c**-**f** The GSEA of ZFPM2-AS1

The lncRNA expression signatures of HCC were investigated based on 50 pairs of HCC tissues and adjacent normal tissues. The results of differential expression analysis of ZFPM2-AS1(ENSG00000251003) in HCC showed that the expression of ZFPM2-AS1 in tumor tissues increased significantly in both matched (Fig. 2a) and unmatched (Fig. 2b) tissues compared to normal tissues (Table S4). Then, we examined the relationship between ZFPM2-AS1 and clinical characteristics. The results (Table 1) showed that the expression level of ZFPM2-AS1 was significantly correlated with the age and survival status of patients. Further multivariate Cox survival analysis showed that the ZFPM2-AS1 expression level was the only independent factor that could affect patient survival (Table 2).

**Table 1:** Association between clinicopathological parameters and ZFPM2-AS1 gene expression in HCC patients

	ZFPM2-AS1 gene expression		Total(n)	***P***-value
	Low(n)	High(n)		
**Age**				
≤ 60	76	55	131	0.009*
>60	43	63	106	
**Gender**				
Female	41	34	75	0.403
Male	78	84	162	
**T classificaion**				
T1 + T2	87	82	169	0.568
T3 + T4	32	36	68	
**N classificaion**				
N0	117	116	233	1
N1 + N2	2	2	4	
**M classificaion**				
M0	117	116	233	1
M1	2	2	4	
**Stage classificaion**				
Stage I + II	85	80	165	0.574
Stage III + IV	34	38	72	
**Survival status**				
Live	90	71	161	0.012*
Dead	29	47	76	

^*^P < 0.05

**Table 2:** Univariate and multivariate analyses of individual parameters for correlations with overall survival rate: Cox proportional hazards model

Characteristics	Univariate Cox				Multivariate Cox			
	p-Value	Hazard Ratio	95% CI		p-Value	Hazard Ratio	95% CI	
			Lower	Upper			Lower	Upper
**Gender**	0.245	0.759	0.478	1.207				
**Age**	0.379	1.225	0.779	1.924				
**T classificaion**	<0.001*	3.094	1.966	4.87	0.638	1.615	0.22	11.873
**N classificaion**	0.305	2.092	0.511	8.562				
**M classificaion**	0.02*	3.973	1.246	12.676	0.411	1.65	0.5	5.443
**Stage**	<0.001*	3.076	1.955	4.839	0.561	1.804	0.246	13.2
**ZFPM2-AS1 gene expression**	0.008*	1.882	1.183	2.993	0.024*	1.716	1.073	2.743

*Abbreviation*: *HR* Hazard Ratio, *CI* Confidence Interval

^*^Statistically significant (*p* < 0.05)

Finally, gene set enrichment analysis (GSEA) of a single gene was performed to determine the molecular function of ZFPM2-AS1, and the results (Fig. 2c-f) showed that ZFPM2-AS1 was closely related to proliferation, apoptosis and related pathways, such as mTOR, PI3K/AKT and reactive oxygen species.

### Knockdown of lncRNA ZFPM2-AS1 inhibited cell proliferation, migration and invasion and promoted cell apoptosis in vitro

**Fig. 3:**
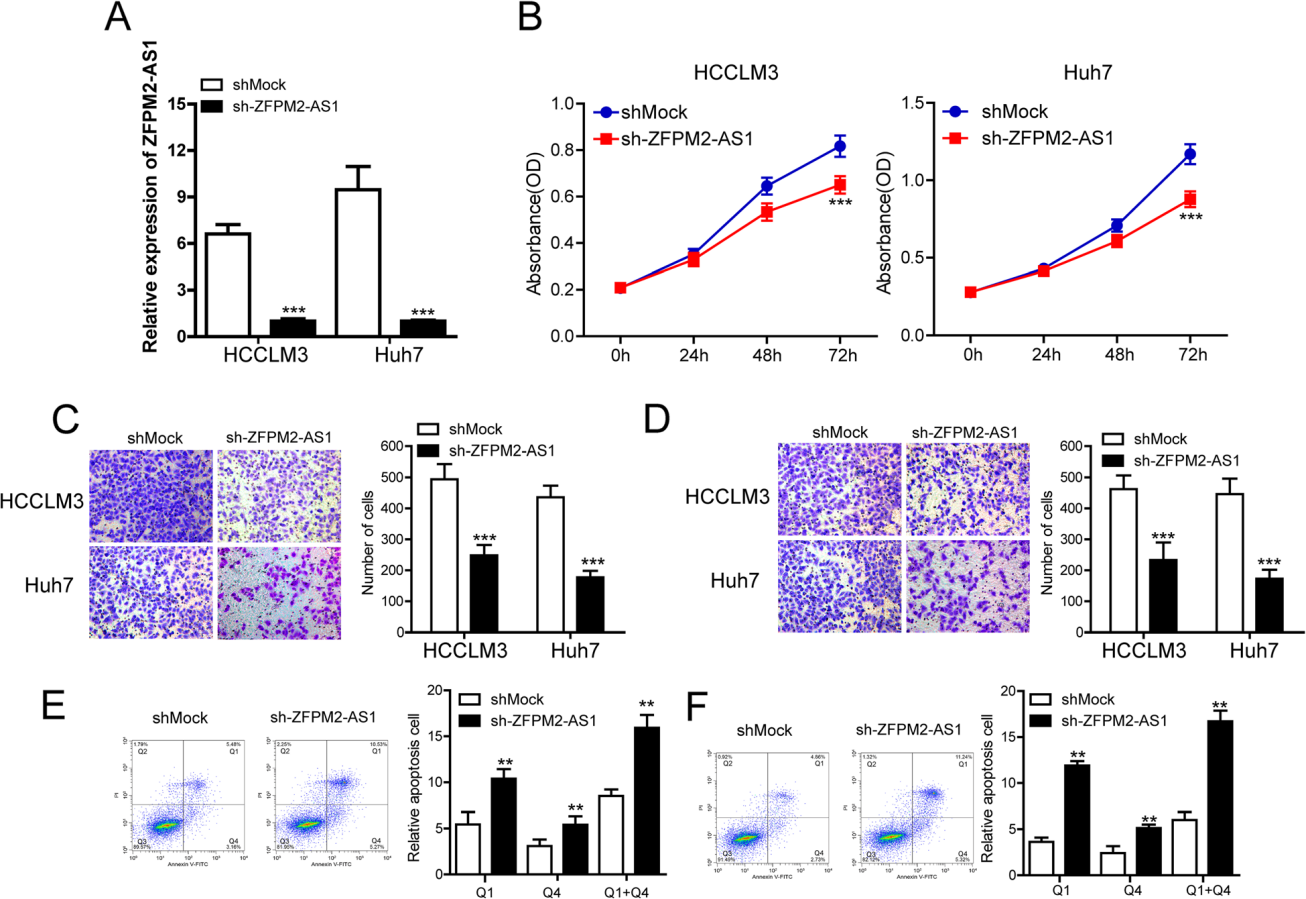
Knockdown of lncRNA ZFPM2-AS1 inhibited cell proliferation, migration and invasion and promoted cell apoptosis in vitro. **a** The regulatory effect of short-hairpin ZFPM2-AS1 (sh-ZFPM2-AS1) transfection on the level of ZFPM2-AS1 in HCC cell lines (Data are expressed as the mean ± standard deviation, *** p < 0.001). **b** Effect of lncRNA ZFPM2-AS1 silencing on viability of HCC cell lines (Data are expressed as the mean ± standard deviation, *** *p* < 0.001). **c** and **d** Knockdown of lncRNA ZFPM2-AS1 significantly inhibited migration (**c**) and invasion (**d**) of HCC cells (Data are expressed as the mean ± standard deviation, *** *p* < 0.001). **e** Knockdown of lncRNA ZFPM2-AS1 significantly induced apoptosis of HCC cells (Data are expressed as the mean ± standard deviation, ** *p* < 0.01)

Considering that lncRNA ZFPM2-AS1 was upregulated in HCC tissues, we next investigated the effects of lncRNA ZFPM2-AS1 silencing on HCC cell phenotypes. Because the expression of ZFPM2-AS1 was relatively high in HCCLM3 and Huh7 cells, we performed short-hairpin ZFPM2-AS1 (sh-ZFPM2-AS1) transfection in these two cell lines and found that the expression was effectively downregulated compared with that in the mock control (shMock) (Fig. 3a). Intriguingly, lncRNA ZFPM2-AS1 silencing markedly inhibited cell viability in both HCCLM3 and Huh7 cells (Fig. 3b). Moreover, the migration and invasion abilities in both HCC cell lines were also significantly suppressed by lncRNA ZFPM2-AS1 silencing (Fig. 3c and d). Flow cytometry analysis demonstrated that compared with shMock, sh-ZFPM2-AS1 strongly increased apoptosis in HCC cells (Fig. 3e and f).

### LncRNA ZFPM2-AS1 acts AS a ceRNA in the regulation of GDF10 expression by binding to miR-139

**Fig. 4:**
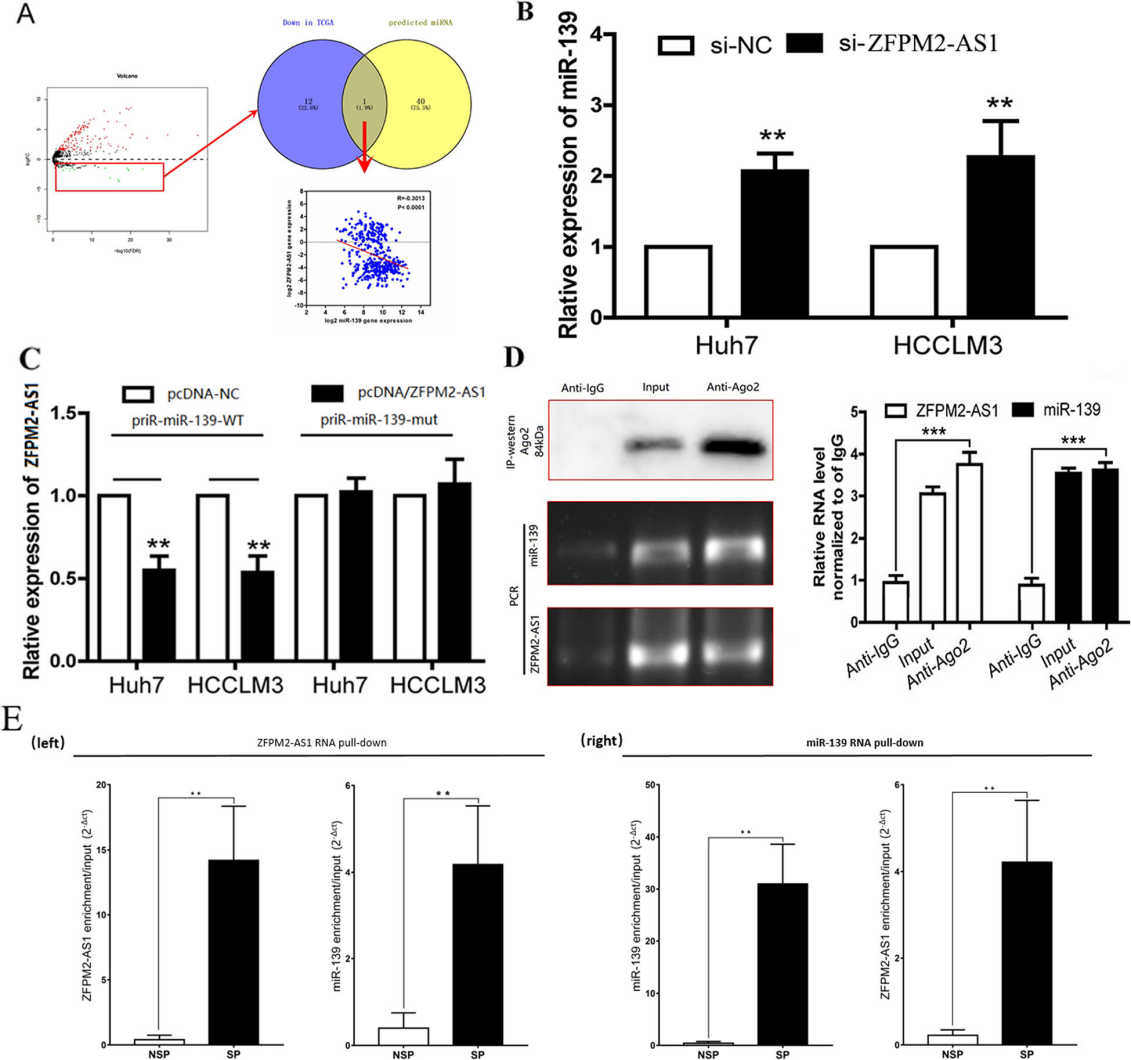
Bioinformatics analysis, dual luciferase assay and RIP experiments confirmed the interaction of ZFPM2-AS1 with miR-139. **a** Bioinformatics analysis predicted that ZFPM2-AS1 has a negative correlation with miR-139. **b** PCR detection of the expression levels of miR-139 in wild type or ZFPM2-AS1 silenced Huh7 and HCCLM3 cells. **c** Luciferase reporter assay to detect the luciferase activity of pmiR-ZFPM2-AS1 in Huh7 and HCCLM3 cells cotransfected with miR-139-mut/wt. **d** IP-western detection of anti-Ago2 antibody capture in HCCLM3 cell lysates, with IgG as the negative control and Input as the positive control. PCR and qRT-PCR was both used to detect the expression of ZFPM2-AS1 and miR-139 in the immunoprecipitated and the same expression trends were observed. IgG was the negative control and Input was the positive control. Data are expressed as the mean ± standard deviation, ** *p* < 0.01, *** *p* < 0.001. (**e**_left) qPCR validation of ZFPM2-AS1 and miR-139 enrichment versus input after ZFPM2-AS1 RNA pull-down by specific probes (SP) as compared to non-specific probes (NSP) in HCCLM3 cells. (**e**_right) qPCR validation of miR-139 and ZFPM2-AS1 enrichment versus input after miR-139 RNA pull-down by specific probes (SP) as compared to non-specific probes (NSP) in HCCLM3 cells(**p < 0.01)

To identify the potential miRNA targets of lncRNAZFPM2-AS1, in silico analysis was performed by using the Microcode database, and the results showed that multiple miRNAs may act as biological targets of lncRNA ZFPM2-AS1 (the results are shown in the table “microRNA prediction-microRNACode.xlxs” and Fig. 4a). It is expected that as the expression of ZFPM2-AS1 in HCC increases, the expression level of the specific downstream miRNA of ZFPM2-AS1 is likely to be downregulated. By analyzing the differentially expressed miRNAs in HCC among paired samples, it was found that there were 13 miRNAs whose expression levels were significantly downregulated in tumor tissue. Astoundingly, only one (miR-139) of them was a potential miRNA target of lncRNA ZFPM2-AS1 in the above in silico analysis. The co expression analysis showed that there was a significant correlation between the expression of miR-139 and ZFPM2-AS1, and the correlation coefficient was − 0.3013, as shown in Fig. 4a. The expression levels of miR-139 were significantly upregulated in HCC cells by silencing ZFPM2-AS1 (Fig. 4b). In addition, ZFPM2-AS1 overexpression markedly decreased luciferase activity in miR-139-wild type but not in miR-139-mut in both Huh7 and HCCLM3 cell lines (Fig. 4c). Subsequent RIP assays detected the expression of ZFPM2-AS1 and miR-139 in the immunoprecipitate by qRT-PCR (Fig. 4d) and showed that both ZFPM2-AS1 and miR-139 could bind tightly to the Ago2 protein. All the above experiments confirmed that miR-139 is a target of lncRNA ZFPM2-AS1 in HCC.

We conducted RNA pull-down assays in HCCLM3 cells to test the interactome between miR-139 and ZFPM2-AS1. The relative enrichment in miR-139 or ZFPM2-AS1 was calculated for non-specific (NSP) or specific probes (SP) relative to the input samples by qRT-PCR. Results showed the specific probes could efficiently to pull-down miR-139 and ZFPM2-AS1 compared with non-specific probes in HCCLM3 cell lysis. After ZFPM2-AS1 RNA pull-down, miR-139 was found to be targeted by ZFPM2-AS1. Reciprocally, ZFPM2-AS1 was significantly enriched after miR-139 RNA pull-down (Fig. 4e).

### Effect of ZFPM2-AS1 on the biological function of miR-139 in HCC cells

**Fig. 5:**
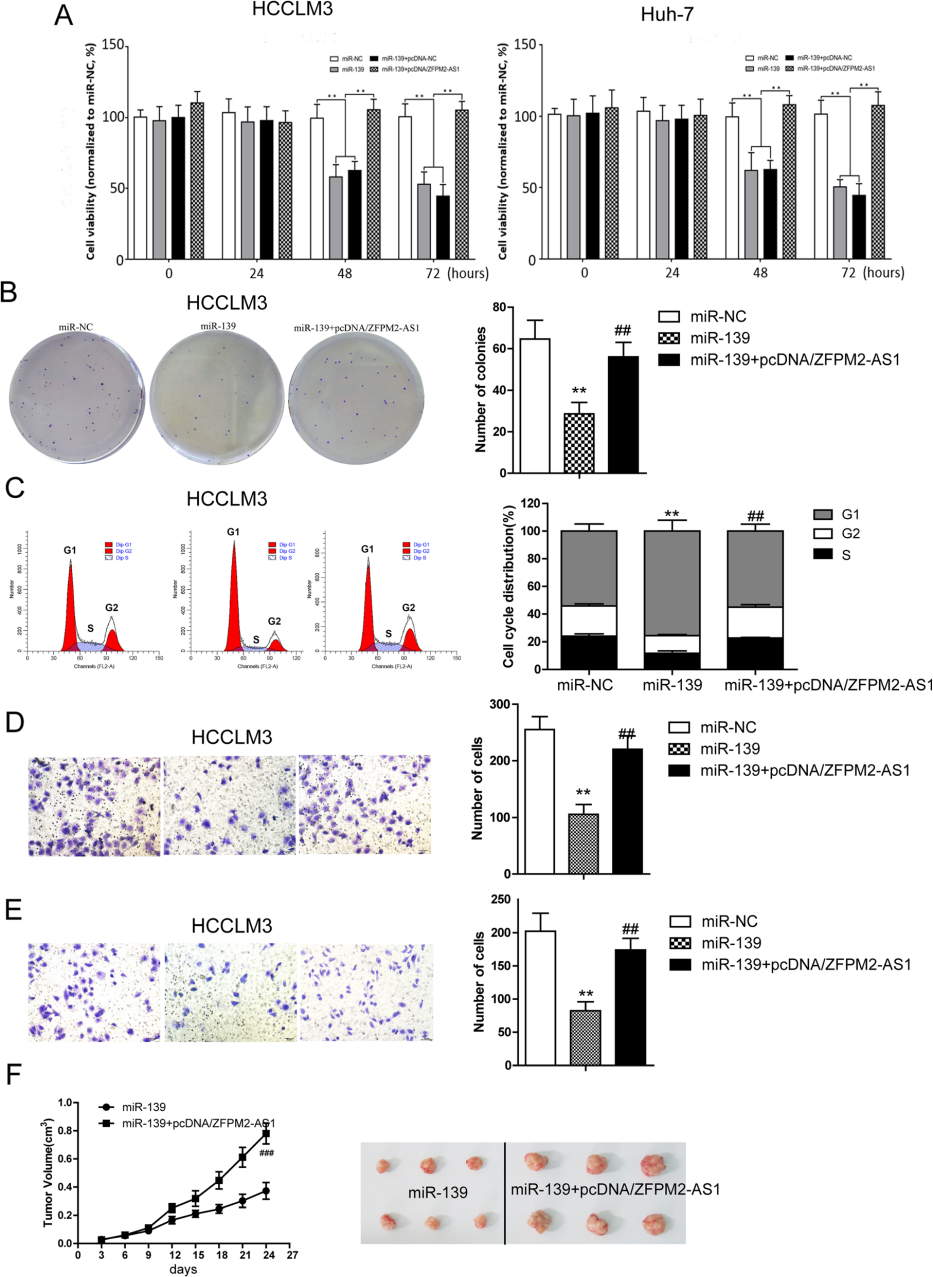
Effect of ZFPM2-AS1 on the biological function of miR-139 in HCC cells. **a** CCK-8 assay of Huh7 and HCCLM3 cells transfected with miR-NC, miR-139, miR-139 + pcDNA-NC and miR-139 + pcDNA/ZFPM2-AS1 at 0 h, 24 h, 48 h and 72 h to measure cell viability. **b** Colony formation assay to detect the colony formation ability of HCCLM3 cells transfected with miR-NC, miR-139 and miR-139 + pcDNA/ZFPM2-AS1.The right side is the number of cell clones. **c** Flow cytometry was used to detect the cell cycle distribution of miR-NC-, miR-139- and miR-139 + pcDNA/ZFPM2-AS1-transfected HCCLM3 cells, and the right side is the cell cycle distribution. **d**-**e** Transwell chamber assay was used to detect the migration (**d**) and invasion (**e**) ability of miR-NC-, miR-139- and miR-139 + pcDNA/ZFPM2-AS1-transfected HCCLM3 cells. The right side is a statistical graph of the migrating cell count and the invasive cell count, respectively. **f** Huh7 cell xenograft tumor growth curve. miR-139- and miR-139 + pcDNA/4 ZFPM2-AS1-transfected Huh7 cells were injected into the axilla of nude mice for 6 weeks, and the nude mice were dissected for tumor tissue. Statistical data are expressed as the mean ± standard deviation, **P < 0.01 (vs miR-NC), ^##^P < 0.01, ^###^*P* < 0.001 (vs miR-139)

Given that the above findings confirmed that miR-139 may be a potential target for ZFPM2-AS1, we further analyzed the effect of ZFPM2-AS1 on miR-139 function. Compared with the miR-NC group, transfection of miR-139 significantly inhibited the viability of HCCLM3 and Huh7 cells, whereas co transfection with ZFPM2-AS1 significantly reversed the inhibitory effect of the miRNA on cell viability observed with miR-139 alone and miR-139 + pcDNA-NC and restored viability to the original level (Fig. 5a). Subsequent HCCLM3 cell colony formation assays (Fig. 5b) and cell cycle assays (Fig. 5c) also showed similar results. ZFPM2-AS1 reversed the inhibition on cell colony formation and G0/G1 cell cycle arrest caused by miR-139 transfection. These results suggest that miR-139, as a tumor suppressor miRNA, can inhibit HCC cell viability and colony formation and induce cell cycle arrest and that ZFPM2-AS1, as an upstream molecule, can reverse the effect of miR-139.

In addition, we also found that miR-139 transfection significantly inhibited cancer cell invasion (Fig. 5d) and migration (Fig. 5e) compared to miR-NC, but co transfection with pcDNA/ZFPM2-AS1 recovered the invasive ability of HCCLM3 cells. These results suggest that miR-139 can inhibit the invasion and metastasis of HCC cells and that ZFPM2-AS1, as its upstream molecule, can reverse the effect of miR-139.

Finally, in vivo nude mice xenograft experiments also showed that the tumor growth rate of Huh7 cells cotransfectedwithmiR-139 + pcDNA/ZFPM2-AS1 was significantly faster than that of cells transfected with miR-139 alone (Fig. 5f). These findings suggestthat miR-139 can inhibit the growth of HCC cells in nude mice, and ZFPM2-AS1 can restore their in vivo growth potential.

### ZFPM2-AS1 regulates the expression of the miR-139 target gene GDF10

**Fig. 6:**
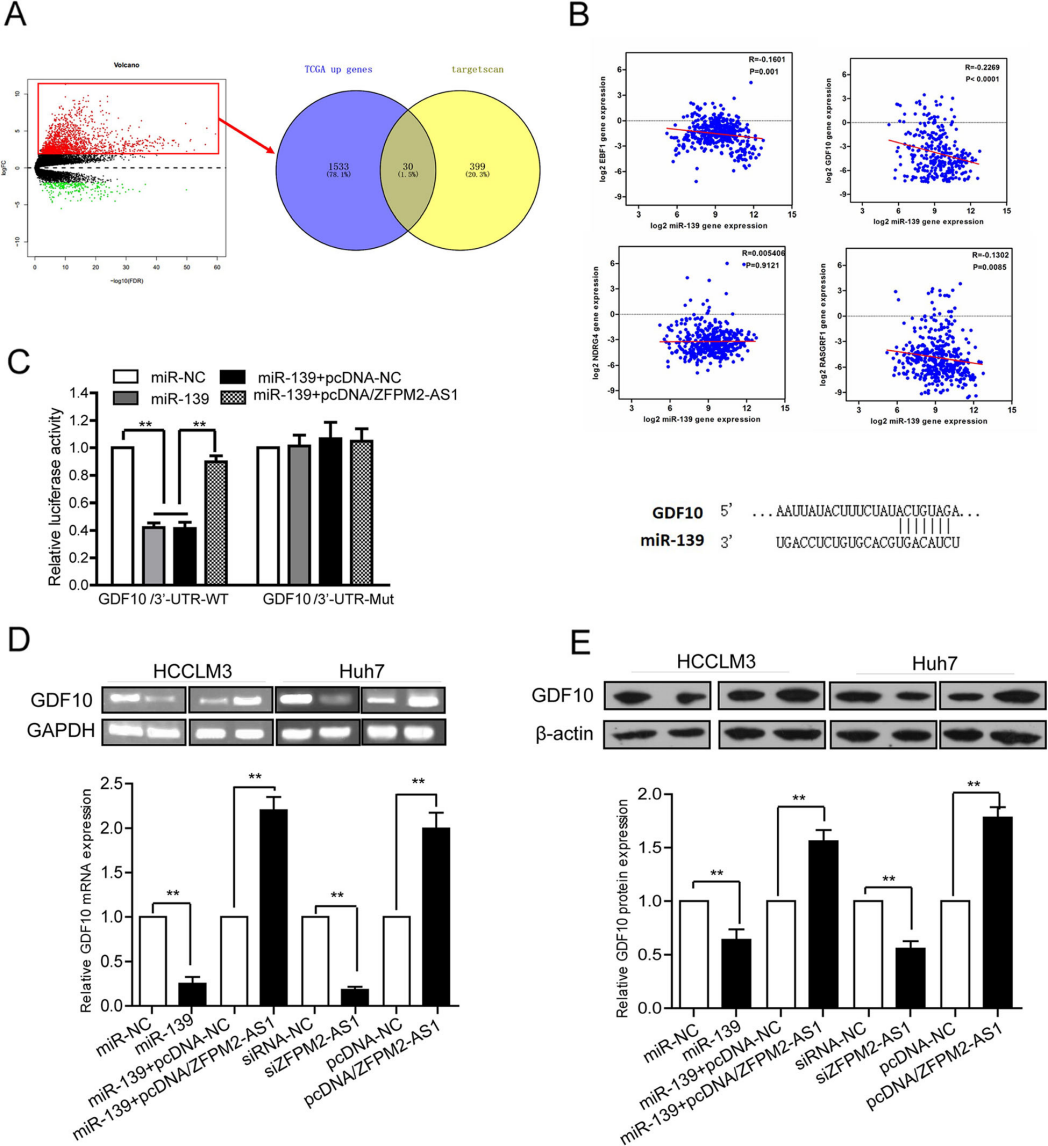
ZFPM2-AS1 regulates the expression of the miR-139 target gene GDF10. **a**-**b** Bioinformatics analysis predicted that GDF10 has a negative correlation with miR-139. The predicted miR-139 binding site in GDF10 were also displayed. **c** Luciferase reporter assay for the detection of pMIR-GDF10 /3′-UTR-WT and pMIR GDF10/3’-UTR-Mutluciferase activity cotransfection with miR-139, miR-NC, miR-139 + pcDNA-NC or miR-139 + pcDNA/ZFPM2-AS1 in Huh7 cells. **d** and **e** The expression of GDF10 mRNA and protein was detected by PCR (**d**) and western blotting (**e**) after transfection of Huh7 and HCCLM3 cells. GAPDH and β-actin were used as internal controls. The data are expressed as the mean ± standard deviation, **P < 0.01

ZFPM2-AS1 can inhibit the biological functions of miR-139, and a question remained whether ZFPM2-AS1 can regulate the expression of target genes of miR-139. First, prediction of target genes of miR-139 was performed by TargetScan, and 430 downstream target genes were obtained (shown in “TargetScan7.1__miR-139-5p.predicted_targets.xlsx” and Fig. 6a). It is expected that as the expression of miR-139 in HCC decreases, the expression level of the specific downstream mRNA of miR-139 is likely to be upregulated. By analyzing the differentially expressed mRNAs in HCC among paired samples, it was found that there were 30 mRNAs whose expression levels were significantly upregulated in tumor tissue (Fig. 6a). Among them, GDF10 was selected as the target gene of miR-139 because it had the highest score in the prediction analysis. The co expression analysis showed that there was a significant correlation between the expression of miR-139 and GDF10 (Fig. 6b_above). The predicted miR-139 binding site in GDF10 is indicated in Fig. 6b_below.

Subsequently, we used a dual luciferase reporter assay to validate the bioinformatics predictions by first constructing GDF10 3′-UTR-WT (wild type) and binding site sequence mutated3′-UTR-Mut (mutant) vectors. The reporter vector pMIR-Report Luciferase was used to observe the effects of miR-139 and ZFPM2-AS1 on luciferase activity in Huh7 cells. The results showed that luciferase activity was significantly decreased in Huh7 cells co transfected with the GDF10 3′-UTR-WT vector and miR-139 compared to cells cotransfected miR-NC. GDF10 was confirmed to be a target gene of miR-139 (Fig. 6c). Furthermore, cells were cotransfected with the GDF10 3′-UTR-WT vector and miR-139 + pcDNA/ZFPM2-AS1, miR-139 aloneormiR-139 + pcDNA-NC. Luciferase activity was partially restored by overexpression of ZFPM2-AS1, *P* < 0.01. The difference between the above groups was not observed in GDF10 3′-UTR-Mut-transfected cells, *P* > 0.05 (see Fig. 6c). The results suggest that ZFPM2-AS1 can bind to miR-139 as a ceRNA and release the binding of miR-139 to the GDF10 3′-UTR.

Subsequent qRT PCR and western blotting further analyzed the effect of miR-139 and ZFPM2-AS1 on the expression of GDF10 in HCC cells. Compared with the miR-NC or siRNA-NC group, miR-139-transfected Huh7 cells and si-ZFPM2-AS1-transfected HCCLM3 cells had significantly decreased expression of GDF10 mRNA and protein (P < 0.01) (Fig. 6d and e). Compared with that in the pcDNA-NC group, the expression of GDF10 mRNA and protein in pcDNA/ZFPM2-AS1-transfected HCCLM3 cells was significantly increased, P < 0.01 (Fig. 6d and e). In addition, compared with those in the miR-139 + pcDNA-NC group, GDF10 mRNA and protein levels were restored in Huh7 cells cotransfected with miR-139 and pcDNA ZFPM2-AS1 (Fig. 6d and e). These findings suggest that ZFPM2-AS1 binds to miR-139 and acts as a ceRNA to prevent miR-139 from inhibiting its target gene GDF10, thereby regulating the expression of GDF10 at the posttranscriptional level.

### miR-139 mimics and si-GDF10 reversed the promoting function of lncRNA ZFPM2-AS1 in vitro

**Fig. 7:**
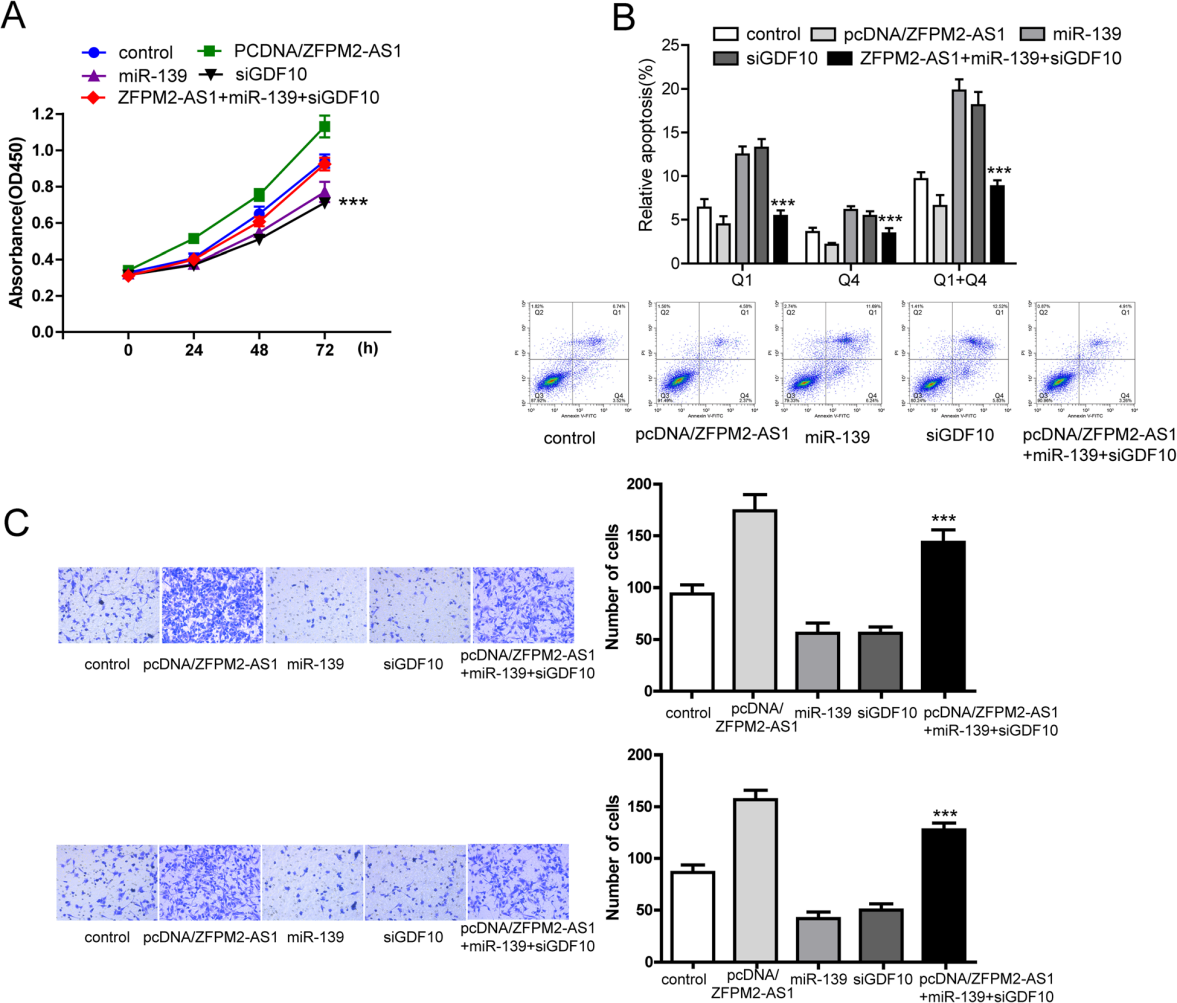
miR-139 mimics and si-GDF10 reversed the promoting function of lncRNA ZFPM2-AS1 in vitro. **a** CCK-8 assay of Huh7 cells transfected with miR-139, siGDF10, pcDNA/ZFPM2-AS1 and miR-139 + siGDF10 + pcDNA/ZFPM2-AS1at 0 h, 24 h, 48 h and 72 h to measure cell viability. **b** Flow cytometry was used to detect the cell apoptosis of miR-139-, siGDF10-, pcDNA/ZFPM2-AS1- and miR-139+ siGDF10+ pcDNA/ZFPM2-AS1-transfected Huh7 cells. **c** Transwell chamber assay was used to detect the migration and invasion ability of miR-139-, siGDF10-, pcDNA/ZFPM2-AS1- and miR-139+ siGDF10+ pcDNA/ZFPM2-AS1-transfected Huh7 cells. The right side is a statistical graph of the migrating cell count and the invasive cell count, respectively. The data are expressed as the mean ± standard deviation, **P < 0.01, ***P < 0.001(vs miR-139)

**Fig. 8:**
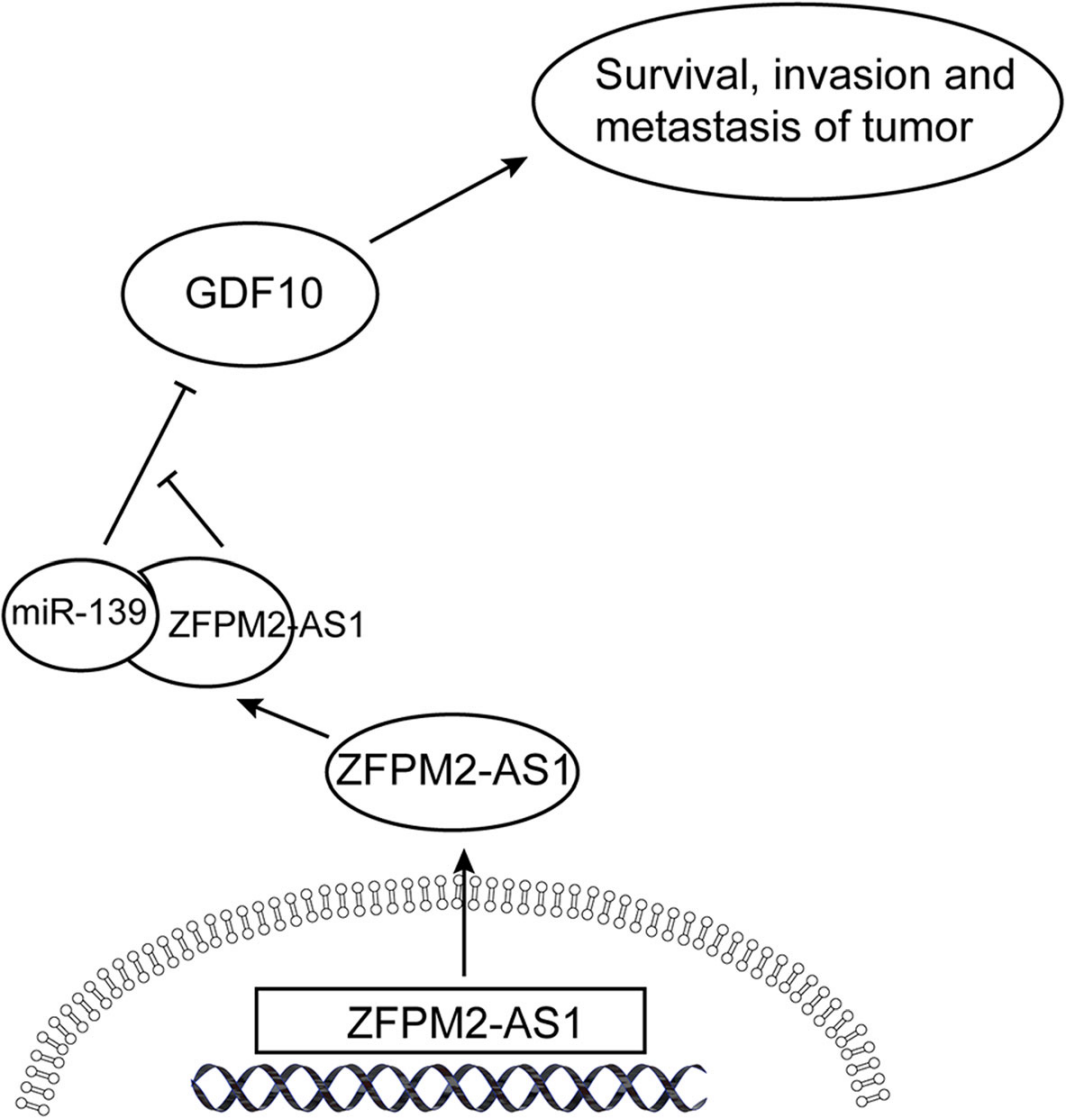
Schematic diagram of ZFPM2-AS1 in HCC progression

Since lncRNA ZFPM2-AS1 acted as a ceRNA in the regulation ofGDF10 expression by binding to miR-139, we performed rescue assays to validate whether miR-139 and GDF10 were involved in the lncRNA ZFPM2-AS1-mediated increase in proliferation, migration and invasion in Huh7 cells. First, we aimed to determine the effects of miR-139 and GDF10 on HCC cell phenotypes. In the rescue experiment, both miR-139 mimics and si-GDF10 remarkably inhibited cell viability, migration and invasion and induced cell apoptosis (Fig. 7). Cotransfection with the ZFPM2-AS1 overexpression vector reversed the decrease in cell viability (Fig. 7a) and the increase in cell apoptosis (Fig. 7b) caused by miR-139 mimics and si-GDF10. Cell migration and invasion experiments revealed that lncRNA ZFPM2-AS1 overexpression markedly reversed the effects of miR-139 mimics and si-GDF10 (Fig. 7c). The schematic diagram in Fig. 8 illustrates the model that ZFPM2-AS1 cooperates with miR139 to inhibit GDF10 and participate in the functional growth of HCC cells.

## Discussion

Early studies on carcinoma genes mainly focused on the differential expression of microRNAs and its role in the occurrence and development of malignancies [19]. MicroRNAs directly affect the proliferation, apoptosis and metastasis of cancer cells by targeting a large number of key protein-coding genes [20]. However, recent studies have found that most of the microRNAs involved in the occurrence and development of cancer have corresponding lncRNAs [21], such as lncRNA H19, NEAT1, MALAT1, which can interact with microRNAs and participate in the occurrence and development of breast cancer [22–24]; lncRNA CASC2 participates in the occurrence of HCC by regulating the microRNA-362-5p/Nf-kappa B axis [25]; HOTAIR as a competitive endogenous RNA interacting with miR-126-5p to promote glioma development [26]; and in lung cancer, lncRNA PVT1–5p promotes cell proliferation by regulating the miR-126/SLC7A5 signaling axis [27]. The regulatory mechanisms of lncRNAs in cancer formation are more diverse and complex than those of miRNAs. There is a specific complex spatial secondary structure within the lncRNA molecule that can provide multiple binding sites to interact with a DNA sequence, RNA sequence, protein or lipid to form a complex and allow lncRNAs to fine tune gene expression within regulatory networks. Recent studies have also shown that lncRNAs can act as a ceRNA or miRNA sponge and compete with miRNAs through their miRNA response elements (MREs) to inhibit the function and activity of miRNAs [28], thereby regulating the expression of the target genes of miRNAs at the posttranscriptional level and participating in the biology of tumor cell proliferation, invasion, metastasis and angiogenesis.

Although a large number of studies have shown that lncRNAs have a certain correlation with the prognosis and some clinical features of HCC patients and may play an important role in the occurrence and development of the disease, the precise molecular mechanism is still not very clear [29–31]. In this study, we first analyzed the differential expression of lncRNAs by analyzing the poorly differentiated and paracancerous samples of HCC patient and their association with patient prognosis. The results showed that ZFPM2-AS1 was significantly up-regulated in the tumor tissues of patients, and its high expression was significantly correlated with the poor prognosis of patients (*p* < 0.05). A single gene GSEA showed that the function of this lncRNA may be related to cell proliferation and apoptosis. Subsequent functional analysis confirmed this prediction, as silencing ZFPM2-AS1 could inhibit cell viability, promote cell apoptosis, and downregulate the ability of cancer cells to invade and migrate. In an earlier study, ZFPM2-AS1 was demonstrated to participate in the occurrence and development of gastric cancer and was positively correlated with depth of tumor invasion, differentiation grade, tumor size, N stage, and TNM stage, suggesting that ZFPM2-AS1 is a potential diagnostic biomarker and/or therapeutic target for gastric cancer. Other studies have shown that the role of ZFPM2-AS1 in the development of gastric cancer is related to its regulation of the ZFPM2-AS1-MIF-p53 signaling axis. Therefore, lncRNA-based lncRNA ZFPM2-AS1-miRNA-mRNA network regulation plays a crucial role in gastric cancer [32]. The expression level of ZFPM2-AS1 in renal tumor tissues was significantly higher than that in corresponding normal counterparts. Analysis of the corresponding clinical characteristics found that the expression of ZFPM2-AS1 is related to lymph node metastasis, tumor stage and survival of renal cell carcinoma patients, further experiments showed that miR-137 is a direct target of ZFPM2-AS1 [33]. Through loss of function analysis, ZFPM2-AS1 deletion would damage the activity of LUAD cells, inhibit cell migration, and reverse the process of epithelial-mesenchymal transition. Besides, ZFPM2-AS1 is involved in the expression of miR-511-3p, thereby deregulating the AF4/FMR2 family member 4 (AFF4) of the miR-511-3p target [34].. Previous research showed that ZFPM2-AS1 was as one of the differential genes in a risk model and demonstrated the potential clinical significance of 7-lncRNA markers for survival prediction in HCC patients [29]. Important remaining questions are whether ZFPM2-AS1 activity affecting patient prognosis in HCC is related to its role as a ceRNA in the regulation of downstream miRNA-mRNA, and what the downstream miRNAs and mRNAs are.

We predicted by the database that there are MREs in the ZFPM2-AS1 sequence that can bind to multiple miRNAs. Based on the differences in the expression of these miRNAs between cancer and adjacent tissues and the correlation with ZFPM2-AS1 expression levels, we selectedmiR-139 for further study. Experiments confirmed that silencing ZFPM2-AS1 in HCC cells significantly upregulated the expression of miR-139. The above results all suggest that there is a certain relationship between ZFPM2-AS1 and miR-139. Furthermore, we confirmed by dual luciferase assay that ZFPM2-AS1 can bind to miR-139 by its MRE. Furthermore, it is well known that miRNAs regulate the expression of target genes by forming an RNA-induced silencing complex (RISC), and recent studies have shown that lncRNAs act as miRNA sponges to regulate miRNA activity and form RISC [35]. Therefore, we used the RIP assay to verify whether ZFPM2-AS1 and miR-139 can bind to RISC. Based on the Ago2 protein being a key protein in the RISC complex [36], we mixed Huh7 cell lysate with an anti-Ago2 antibody bound to magnetic beads. The results of qRT-PCR detection of ZFPM2-AS1 and miR-139 expression in the immunoprecipitate suggest that ZFPM2-AS1 and miR-139 can bind to each other in RISC.

Next, we aimed to determine if the expression of ZFPM2-AS1 affects the biological function of miR-139. Through functional experiments, we found that overexpression of miR-139 could inhibit the proliferation, colony formation, invasion and metastasis of HCC cells, inhibit the growth of xenografts in nude mice, and induce cell cycle G0/G1 phase arrest. In addition, ZFPM2-AS1 overexpression could reverse the inhibitory effect of miR-139 on HCC cell growth and metastasis in vivo and in vitro. The above results indicate that ZFPM2-AS1 acts as a ceRNA and inhibits the function and activity of miR-139.

ZFPM2-AS1 can inhibit the biological function of miR-139, so we set out to characterize the mechanism and determine whether it is related to the expression level of the target gene that regulates miR-139 and also identify the target gene. In this part of the study, we first predicted through bioinformatics analysis that EBF1, NDRG4, RASGRF1 and GDF10 (*P* < 0.0001) are some of the possible target genes ofmiR-139. Based on the differences in the expression of these target genes in cancer and paracancerous tissues and the correlation between the expression levels of ZFPM2-AS1 and miR-139, we focused on GDF10 (protein and mRNA expression was significantly associated with ZFPM2-AS1 expression, with positive correlation, r = 0.7114 and 0.6116, P < 0.0001; and a significant negative correlation with the expression of miR-139, r = − 0.5040 and − 0.7094, P < 0.0001).

GDF10 gene encodes a secreted ligand of the TGF-beta (transforming growth factor-beta) superfamily of proteins. Ligands of this family bind various TGF-beta receptors leading to recruitment and activation of SMAD family transcription factors that regulate gene expression. The encoded preproprotein is proteolytically processed to generate each subunit of the disulfide-linked homodimer. This promotes neural repair after stroke. Additionally, this protein may act as a tumor suppressor and reduced expression of this gene is associated with oral cancer. Therefore, an increasing number of studies have used GDF10 as a target for screening anticancer drugs. Further dual luciferase assays, as well as PCR and WB, confirmed that miR-139 decreased the luciferase activity of GDF10/3′-UTR and the mRNA and protein expression levels of GDF10, whereas overexpression of ZFPM2-AS1 reversed this phenomenon. This result indicates that GDF10 is a target gene of miR-139, and ZFPM2-AS1 can bind to miR-139 as a ceRNA and release the binding of miR-139 to GDF10, thereby regulating the expression of GDF10 at the posttranscriptional level. Finally, rescue assays showed that miR-139 and GDF10 were involved in the lncRNA ZFPM2-AS1-mediated increase in the proliferation, migration and invasion in HCC cells. It was confirmed that the expression patterns and interactions of miR-139 and GDF10 have important biological significance in the occurrence and development of HCC.

## Conclusions

Based on the above findings, ZFPM2-AS1 can act as an oncogene to promote HCC cell proliferation, invasion and metastasis, and the underlying mechanism is mediated by the ZFPM2-AS1-miR-139-GDF10 axis. This finding not only improves our understanding of the molecular mechanism of HCC occurrence and development but also provides a new direction for the diagnosis and treatment of HCC by targeting lncRNA.

## Supplementary information

**Additional file 1.**

**Additional file 2.**

**Additional file 3.**

**Additional file 4.**

**Additional file 5.**

**Additional file 6.**

## Data Availability

The dataset used and/or analyzed during the current study are available from the corresponding author on reasonable request.

## Abbreviations

lncRNAs: long noncoding RNAs
HCC: Hepatocellular carcinoma
TCGA: The Cancer Genome Atlas
ceRNA: Competing endogenous RNA
PVDF: Polyvinylidene difluoride
HRP: Horseradish peroxidase
ECL: Enhanced chemiluminescence
SD: Standard deviation
GSEA: Gene set enrichment analysis
MREs: MiRNA response elements
RISC: RNA-induced silencing complex
